# Transfection of Infectious RNA and DNA/RNA Layered Vectors of Semliki Forest Virus by the Cell-Penetrating Peptide Based Reagent PepFect6

**DOI:** 10.1371/journal.pone.0069659

**Published:** 2013-07-08

**Authors:** Kalle Pärn, Liane Viru, Taavi Lehto, Nikita Oskolkov, Ülo Langel, Andres Merits

**Affiliations:** 1 Institute of Technology, University of Tartu, Tartu, Estonia; 2 Department of Neurochemistry, Stockholm University, Stockholm, Sweden; Commissariat a l'Energie Atomique(cea), France

## Abstract

Viral vectors have a wide variety of applications ranging from fundamental studies of viruses to therapeutics. Recombinant viral vectors are usually constructed using methods of reverse genetics to obtain the genetic material of the viral vector. The physicochemical properties of DNA and RNA make them unable to access cells by themselves, and they require assistance to achieve intracellular delivery. Non-viral delivery vectors can be used for this purpose if they enable efficient intracellular delivery without interfering with the viral life cycle. In this report, we utilize Semliki Forest virus (genus *alphavirus*) based RNA and DNA vectors to study the transfection efficiency of the non-viral cell-penetrating peptide-based delivery vector PepFect6 in comparison with that of the cationic liposome-based Lipofectamine 2000, and assess their impact on viral replication. The optimal conditions for transfection were determined for both reagents. These results demonstrate, for the first time, the ability of PepFect6 to transport large (13-19 kbp) constructs across the cell membrane. Curiously, DNA molecules delivered using the PepFect6 reagent were found to be transported to the cell nucleus approximately 1.5 hours later than DNA molecules delivered using the Lipofectamine 2000 reagent. Finally, although both PepFect6 and Lipofectamine 2000 reagents can be used for alphavirus research, PepFect6 is preferred because it does not induce changes in the normal cellular phenotype and it does not affect the normal replication-infection cycle of viruses in previously transfected cells.

## Introduction

Viral vectors are commonly used when high transduction efficiencies and/or high levels of foreign gene expression are required [1,2]. In addition to foreign gene expression, viral vectors enable the creation of triggered systems for applications where only localized infection is required, such as tumor therapy [3,4]. Full-length viral vectors (also termed replication competent vectors) contain all the characteristic genetic elements of infectious viruses and generally have the ability to spread the infection from the original host cell. This property separates them from other viral and non-viral vectors and creates a potential biological threat to the host.

Semliki Forest virus (SFV) belongs to the genus *Alphavirus* in the *Togaviridae* family and has a relatively small (11.5 kb) single stranded RNA genome with a positive polarity [5]. SFV is capable of infecting various cell types and successfully replicating within those cells. The ability of some strains of SFV (L10, SFV4) to cause encephalitis in rodents allows the virus to be used as a model system for studies of viral neuropathogenesis [6]. In addition to its broad tropism, the SFV has several beneficial properties as a potential vector, including high expression levels of viral subgenomic (SG) mRNA synthesized in infected cells. In the case of wild type SFV, this allows for the expression of viral structural proteins at very high levels; however, as structural proteins are not required for SFV replication, the corresponding part of the viral genome may also be substituted with other sequences of interest. Additional benefits of the SFV as a vector include its small genome, which can be modified with ease using corresponding cDNA clones, and the ability of the viral RNA to induce a productive infection [5].

**Figure 1:**
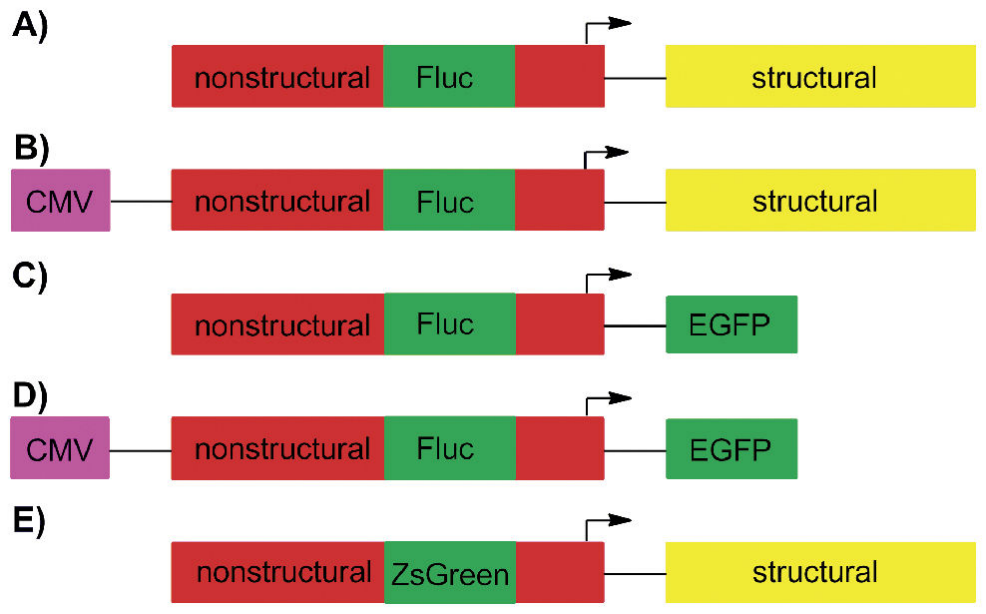
SFV based vectors used in this study. (A) SFV(Fluc) 4, a replication-competent RNA vector containing the Fluc marker in its nonstructural region; (B) pCMV-SFV(Fluc) 4, corresponding DNA/RNA layered vector; (C, D) Replicon vectors SFV(Fluc) 1-EGFP and pCMV-SFV(Fluc) 1-EGFP where the structural region required for virion formation was replaced by the EGFP sequence; (E) SFV(ZsGreen) 4, a replication competent virus containing the ZsGreen marker in its nonstructural region. CMV-cytomegalovirus immediate early promoter. SG promoter of SFV is indicated by an arrow; plasmid backbones of DNA/RNA layered vectors (B, D) are not shown.

The main types of SFV-based vectors include the full-length genomic RNA vector for which the RNA is synthesized on the template of corresponding cDNA by *in vitro* transcription using the RNA polymerase of SP6 bacteriophage [7], the DNA/RNA layered vector where the cDNA copy of the viral genome is placed under the control of cytomegalovirus immediately early promoter to allow for its transcription in the nucleus of the cell [8], and replicon vectors that are obtained through the removal of the region encoding for structural proteins (Figure 1), making the vector unable to form virions and exit the cell [9]. Thorough research is required for the therapeutic application of any of these vectors. Factors that must be taken into account include the ability of vectors to replicate under various conditions, their genetic stability, their ability to express the desired foreign gene(s) and their potential to induce *in vivo* pathogenesis.

The cDNAs and genomes of recombinant viral vectors are usually generated by means of recombinant DNA technology [10]. In several cases, obtained viral genomes or DNA/RNA layered vectors can also be used as therapeutic tools [11]. Unlike virions, such materials are not able to enter the cells by themselves. Therefore, efficient non-viral transfection vectors and/or other methods that would help to overcome this obstacle are needed. In addition, delivery of the genetic material into the cell should not inhibit the subsequent replication cycle of the vector. Unfortunately, not all available transfection systems meet these criteria, requiring thorough research on how different transfection methods work for constructs based on viral nucleic acids. Non-viral transfection reagents are usually based on different cationic polymers [12], lipids [13] or peptides [14] that have the ability to condense molecules of nucleic acids into nano-sized particles or allow chemical conjugation between these entities, facilitating the transport of nucleic acids into the cells. Common problems with non-viral delivery of viral materials include low efficiency of transfection and various side effects including the direct inhibition (or, in some cases, boosting) of viral replication and/or the activation of antiviral cellular responses.

**Figure 2:**
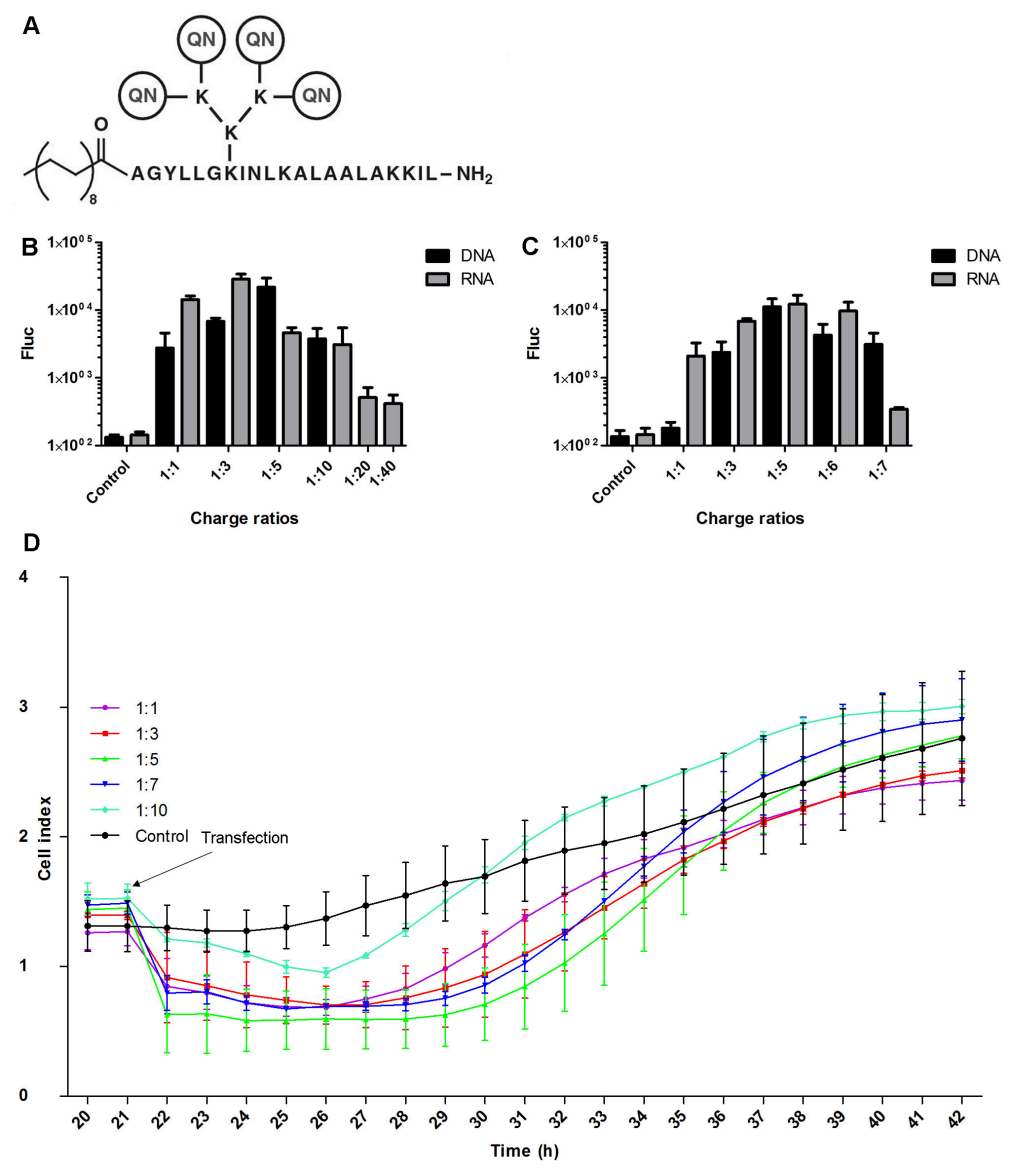
Optimization of the nucleic acid: PF6 charge ratios for cell transfection. (A) Structure of the PF6 reagent. QN-trifluoromethylquinoline moiety. (B) BHK-21 and (C) MEF cells grown in a 24-well cell culture plate were transfected using 1 µg of pCMV-SFV(Fluc) 4 (DNA) or SFV(Fluc) 4 (RNA) and different amounts of PF6 reagent. Fluc activities were measured 8 h post transfection for BHK-21 cells and 24 h post transfection for MEF cells. Fluc activities, shown on the vertical axes, are normalized to the amount of total protein. This experiment was performed in triplicate, and the error bars indicate standard deviations. (D) Analysis of the effects of PF_6_^-^based transfection mixtures on the cell growth measured using the xCELLigence System. BHK-21 cells were plated on the E-plate and grown for 21 h. At this time point, transfection mixtures consisting of the pSFV(Fluc) 4 plasmid and the PF6 reagent at charge ratios of 1:1, 1:3, 1:5, 1:7 or 1:10 were added; the final concentration of the DNA in the transfection media was the same as in the experiments shown at panels B and C. Growth of transfected cells (cell index) was monitored up to 22 h post transfection. This experiment was performed in duplicate, and the error bars indicate standard deviations.

One class of non-viral transfection reagents is cell-penetrating peptides (CPPs), short peptide that have been shown to be efficient vectors for the delivery of nucleic acids both *in vitro* and *in vivo* [15–17]. Recently, we have developed a novel group of chemically modified CPP-based vectors named PepFects [18], which are compatible with the delivery of nucleic acids in nanoparticle form. Among this family, PepFect6 (PF6) (Figure 2A) is based on the transportan 10 peptide but also includes a pH-sensitive endosomolytic modification and a stearic acid moiety, rendering it a highly efficient vehicle for the delivery of short oligonucleotides both *in vitro* and *in vivo* [19]. In the present work we investigated how PF6 and the cationic-lipid based Lipofectamine 2000 (LF2000) reagent could be used for the delivery of DNA and RNA based SFV expression vectors into eukaryotic cells and examined the effects of these transfections on subsequent viral infection. The results presented here reveal for the first time that CPPs are suitable for transfection of cells with *in vitro* transcribed RNAs or DNA/RNA layered plasmids of viral vectors. Infection in cells transfected using PF6: SFV DNA/RNA layered vector complexes began later than in cells transfected with LF2000-based complexes, indicating slower release and/or nuclear transport of PF_6_^−^bound DNA molecules. Nevertheless, the efficiency of CPP-based transfection was comparable with and in some cases superior to that of LF2000. Furthermore, the use of CPPs did not affect subsequent infection of SFV in transfected cells.

## Materials and Methods

### Recombinant viruses and vectors

The RNA genome of SFV(Fluc) 4 contains the coding sequence of firefly luciferase (Fluc) marker inserted into the nonstructural region of the vector (Figure 1A) as previously described by Tamberg et al. [20]. The corresponding DNA/RNA layered vector pCMV-SFV(Fluc) 4 (Figure 1B) was used for infectious plasmid DNA transfection experiments. SFV non-propagating replicon vector experiments used pSFV(Fluc) 1-EGFP, a plasmid that contains the cDNA of the SFV(Fluc) 1-EGFP replicon (expresses Fluc and EGFP markers, Figure 1C) and a DNA/RNA layered vector pCMV-SFV(Fluc) 1-EGFP (Figure 1D) of similar design. Experiments aimed at monitoring SFV replication organelles used SFV(ZsGreen) 4, a virus encoding nonstructural protein (nsP) 3 fused with ZsGreen marker (Figure 1E). *In vitro* transcription of pSFV(Fluc) 4, pSFV(Fluc) 1-EGFP and pSFV(ZsGreen) 4 was carried out using the mMessage mMachine SP6 kit (Ambion). The quality and quantity of the RNAs obtained in this way was assessed using agarose gel electrophoresis.

Complete sequences of all these vectors are available from the authors upon request.

Cell lines and growth conditions. Baby hamster kidney cells (BHK-21; ATCC-CCL-10) were grown at 37°C, 5% CO_2_ in Glasgow Modified Eagle’s Medium (GMEM, Gibco) supplemented with 200 mM HEPES, pH 7.2, 100 U/ml penicillin, 100 µg/ml streptomycin and 10% Fetal Calf Serum (FCS, PAA). Primary mouse embryonic fibroblast (MEF) cells (CF-1 strain, Millipore) were grown at 37°C, 5% CO_2_ in Dulbecco’s Modified Eagle’s Medium (DMEM, Gibco) supplemented with 10% FCS, 100 U/ml penicillin, 100 µg/ml streptomycin and 0.0007% 2-mercaptoethanol. Before the seeding of the MEF cells, the surface of the cell culture dish was treated with a 0.1% gelatin solution. Chinese hamster ovary (CHO) cells (ATCC-CCL-61) were grown at 37°C, 5% CO_2_ in F-12 medium supplemented with 10% FCS, 100 U/ml penicillin, 100 µg/ml streptomycin, 0.01 mM sodium pyruvate and 0.01 mM of non-essential amino acids.

### Synthesis of PepFect6

The peptide was synthesized in a stepwise manner at a 0.1 mmol scale by an ABI 433A automated peptide synthesizer (Applied Biosystems) using a N-Fmoc (N-fluorenylmethyloxycarbonyl) solid phase peptide synthesis strategy according to a previously reported procedure [19]. The peptide was purified by a preparative HPLC system (Agilent) using a reverse-phase C4 column (Phenomenex Jupiter C4, 5 µm, 300 Å, 250x10 mm) with a gradient of 30–100% acetonitrile/water/0.1% TFA. The molecular mass of the peptide was analyzed by a Voyager-DE PRO MALDI-TOF mass-spectrometer (Applied Biosystems) using α -cyano-4-hydroxycinnamic acid (Sigma Aldrich) as the matrix in positive ion reflector mode. After freeze-drying, the purity of peptide was approximately 95% as determined by analytical HPLC on a C18 column (Agilent, Eclipse XDB-C18, 5 µm, 4.6x150 mm). The molar concentration of the peptide solution was determined based on dilutions of accurately weighed substances.

### The transfection of cells with plasmid DNAs and *in vitro* transcribed RNAs

In all experiments, 1 µg of DNA or RNA was used to transfect 60-70% confluent cells growing on a 2 cm^2^ area (one well of a 24 well cell culture plate). The PF6 reagent was used to deliver plasmid DNAs or *in vitro* synthesized RNA into cells growing in media containing 10% FCS. LF2000 (Invitrogen) was used according to the manufacturer’s protocols.

In optimization experiments, the charge ratios of the DNA (or RNA) to the PF6 reagent were 1:1, 1:3, 1:5, 1:6, 1:7, 1:10, 1:20 or 1:40; the 1:1 ratio corresponds to a final peptide concentration of 10.5 µM, 1:3 corresponds to 31.5 µM, etc. Subsequently, the charge ratios 1:3 for RNA and 1:5 for DNA were used for the transfection of BHK-21 cells, while the charge ratio of 1:5 was used for both DNA and RNA for the transfection of MEF cells. In siRNA transfection experiments, CHO and CHO-EGFP cells were transfected with 10.5 µl 100 nM anti-EGFP siRNA (Ambion) according to the protocol used by El Andaloussi et al. [19]. The transfection mixtures were created by first mixing nucleic acids and water, and then the required amount of transfection reagent was added. The components were gently mixed and incubated at room temperature for 1 h, and then 100 µl of mixture was added to cells pre-washed with fresh growth media prior to transfection. Experiments aiming to optimize the amount of transfection reagent used a 120 min transfection period. In experiments aiming to optimize the length of the transfection period, incubation times ranged from 0 (immediate replacement of transfection media) to 120 min. Based on these results, a 40 min transfection period was used in subsequent experiments. Transfected cells were incubated for the selected period of time and then lysed using 200 µl Cell Culture Lysis Reagent (Promega). Luciferase activity was measured using a Glomax SIS instrument (Promega), and the results were normalized to the amount of total protein in the lysate determined using the Bradford assay.

The effects of PF6 containing transfection mixtures on the growth of transfected cells was analyzed using the xCELLigence System (Roche) and corresponding electrode plates (E-plate). This system makes real time measurements of the electrode impedance, displayed as cell index values. For this assay, BHK-21 cells were plated in a 16-well E-plate and grown for 21 h. At this time point, transfection mixtures consisting of pSFV(Fluc) 4 and PF6 reagent at charge ratios 1:1, 1:3, 1:5, 1:7 or 1:10 were added; the final concentration of the DNA in the transfection media was the same as in the rest of the experiments. Growth of the transfected cells (cell index) was monitored for 22 h.

### Determination of the percentage and morphology of transfected cells

To determine the percentage of transfected cells and their morphology, 60-70% confluent BHK-21 cell cultures were transfected with *in vitro* RNA transcripts of SFV(Fluc) 1-EGFP or with the pCMV-SFV(Fluc) 1-EGFP DNA/RNA layered vector. The EGFP positive and EGFP negative cells were counted 14 h post transfection in 20 fields of view and in three repeats using a Nikon Eclipse TS 100 microscope. The results were averaged and used to calculate the percentage of transfected cells. In addition, Fluc activity in the cell lysates was also measured. To determine the morphology of the transfected cells and the presence of viral replication organelles, cells were grown on glass slips and transfected and incubated as described above. Cells were fixed and immunofluorescence analysis was carried out using antiserum against nsP3 of SFV (guinea pig, in-house) as the primary antibody and Alexa 560 conjugated anti-guinea pig antibody (Invitrogen) as the secondary antibody as previously described by Varjak et al. [21]. Images of these samples were captured using an Olympus FV 1000 microscope.

### Analysis of the release of self-replicating viral RNAs from DNA/RNA layered vectors

BHK-21 cells at 60-70% confluence on a 2 cm^2^ growth area were transfected with pCMV-SFV(Fluc) 1-EGFP as described above, with the exception that actinomycin D (final concentration 20 µg/ml) was added either together with the transfection mixture or 30, 60, 90, 120 or 180 min later. Regardless of the time of addition of actinomycin D, the cells were collected 8 h post transfection and the Fluc activity in the cells was determined as described above.

### Assessment of the effects of initial transfection on subsequent SFV infection

CHO and CHO-EGFP cells grown on coverslips were transfected with siRNAs targeting EGFP mRNA. For the PF6 reagent, the siRNA: PF6 charge ratio was 1:40. Cells were subsequently infected with SFV(ZsGreen) 4 at 2 h, 4 h, 8 h or 24 h post transfection at a multiplicity of infection (MOI) of 0.1 plaque forming units/cell in serum-free growth medium. After 1 h, the infection medium was replaced with normal growth medium; cells were fixed at 4 h or 6 h post infection. Cells were stained using rabbit anti SFV nsP1 serum (in-house) as the primary antibody and Alexa 568 conjugated anti-rabbit antibody (Invitrogen) as the secondary antibody. Nuclei were counter-stained with DAPI and expression of ZsGreen was determined by its green fluorescence. Images of these samples were collected using LSM 710 confocal microscope (Zeiss).

## Results

### Optimization of the transfection parameters

To trigger viral replication, the vector RNA must first reach the cell and should be released to the cytoplasm. If DNA/RNA layered vectors are used, then the DNA must also be transported to the nucleus. To perform these tasks, a suitable method of delivery that would ideally have no adverse effects on virus infection must be chosen and optimized. Therefore, we first determined the optimal charge ratio between viral nucleic acid and PF6 reagent as well as the optimal length of the transfection period for BHK-21 and MEF cells. In these experiments, cells were collected and analyzed at 8 h (BHK-21) or 24 h (MEF) post transfection, with the collection times selected based on previous observations (unpublished results). Longer times were required for MEF cells because these primary cells have intact innate immune systems [22] that make it more difficult for the virus to reach measurable levels of gene expression.

To optimize the transfection conditions, BHK-21 cells were transfected either with 1 µg of *in vitro* synthesized RNA transcript of pSFV(Fluc) 4 or 1 µg of purified pCMV-SFV(Fluc) 4 DNA/RNA layered vector; the ratios between the negative charges in the nucleic acids and the positive charges in PF6 molecules ranged from 1:1 to 1:40. This analysis revealed that the optimal ratio for RNA transfection was 1:3 and that for DNA transfection was 1:5 (Figure 2B); these charge ratios were subsequently used for all experiments with BHK-21 cells. It was also noted that the RNA: PF6 complexes with charge ratios of 1:20 and 1:40 caused rapid lysis of the majority of transfected cells (data not shown); this resulted in a drastic drop in the marker expression by the reporter virus (Figure 2B). Such toxic effects were also observed if the same amounts of PF6 were used without RNA or DNA (data not shown). Therefore, charge ratios of 1:20 or 1:40 cannot be used for the transfection of large RNA or DNA molecules. As cell lysis was not observed at other charge ratios, we used the xCELLigence System to verify whether or not such transfection mixtures affect the growth of transfected cells. In this assay, BHK-21 cells were transfected using mixtures containing the pSFV(Fluc) 4 plasmid, as it is similar in size to the pCMV-SFV(Fluc) 4 but lacks infectivity. Thus, the effects observed for the transfected cells would be caused by transfection rather than by the release of recombinant virus. This experiment revealed that transfection mixtures with charge ratios of 1:1, 1:3, 1:5 and 1:7 all had very similar effects on the measured cell index (Figure 2D). The addition of transfection mixture resulted in an immediate decrease in impedance as highly charged DNA: PF6 complexes increased electrical conductivity. This effect was detectable for approximately 9 h, after which all cell indexes began to increase, in 5-6 h reaching that of control cells (Figure 2D). Thus, transfection with these DNA: PF6 complexes had no effect on cell growth. Somewhat surprisingly, the transfection mixture with a DNA: PF6 charge ratio of 1:10 led to a less prominent decrease in impedance. Nevertheless, at 20 h post transfection, the cell index of this culture was only slightly higher than that of other cultures. Although the causes of this behavior are not known, it is clear that the transfection mixture with a 1:10 charge ratio did not have a negative effect on the growth of BHK-21 cells. We used this knowledge to determine the optimal nucleic acid: PF6 charge ratio for MEF cells; it was found to be 1:5 for both DNA and RNA (Figure 2C). This ratio was also used in all subsequent experiments with MEF cells.

**Figure 3:**
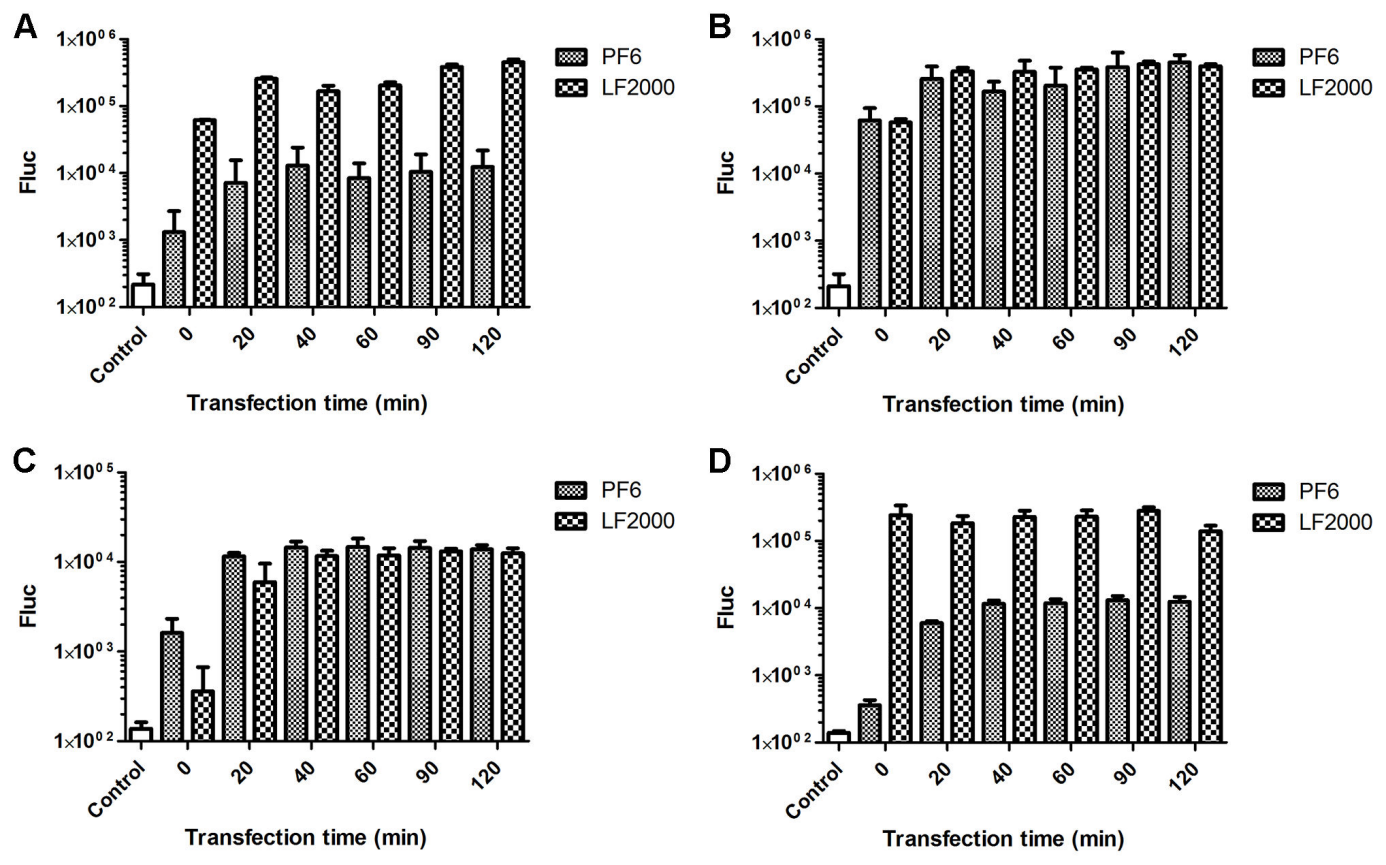
Optimization of transfection times for the PF6 and LF2000 reagents. BHK-21 (A, B) and MEF cells (C, D) were transfected with pCMV-SFV(Fluc) 4 DNA (A, C) or with SFV(Fluc) 4 RNAs (B, D) using PF6 or LF2000 reagents. The transfection time was varied from 0 to 120 min (horizontal axes). Fluc activities were measured 8 h post transfection for BHK-21 cells and 24 h post transfection for MEF cells. Fluc activities, shown on the vertical axes, are normalized to the amount of total protein. This experiment was performed in triplicate, and the error bars indicate standard deviations.

To optimize the duration of transfection, the incubation period of cells with transfection mixture was varied from 0 min to 120 min. For comparison, a commercial LF2000 reagent was used under similar conditions. For both reagents, transfection was observed even if the transfection mixture was immediately replaced with growth medium. For the PF6 reagent, this is in accordance with results by Lee and Pardridge indicating that peptide complexes can be internalized extremely rapidly [23]. However, the optimal transfection levels for both reagents were achieved when 20 min incubation periods were used; longer incubation times did not result in significant increases in transfection efficiency (Figure 3A–D). The head to head comparison of transfection efficiencies showed that LF2000 was more efficient in transfecting BHK-21 cells with DNA vectors (Figure 3A) and MEF cells with *in vitro* RNA transcripts (Figure 3D), while for the transfection of BHK-21 cells with *in vitro* RNA transcripts (Figure 3B) and for the transfection of MEF cells with DNA vectors (Figure 3C), the reagents exhibited highly similar efficiencies.

### PF6 is more efficient for DNA transfection but leads to a delay in the nuclear entry of delivered DNA

**Figure 4:**
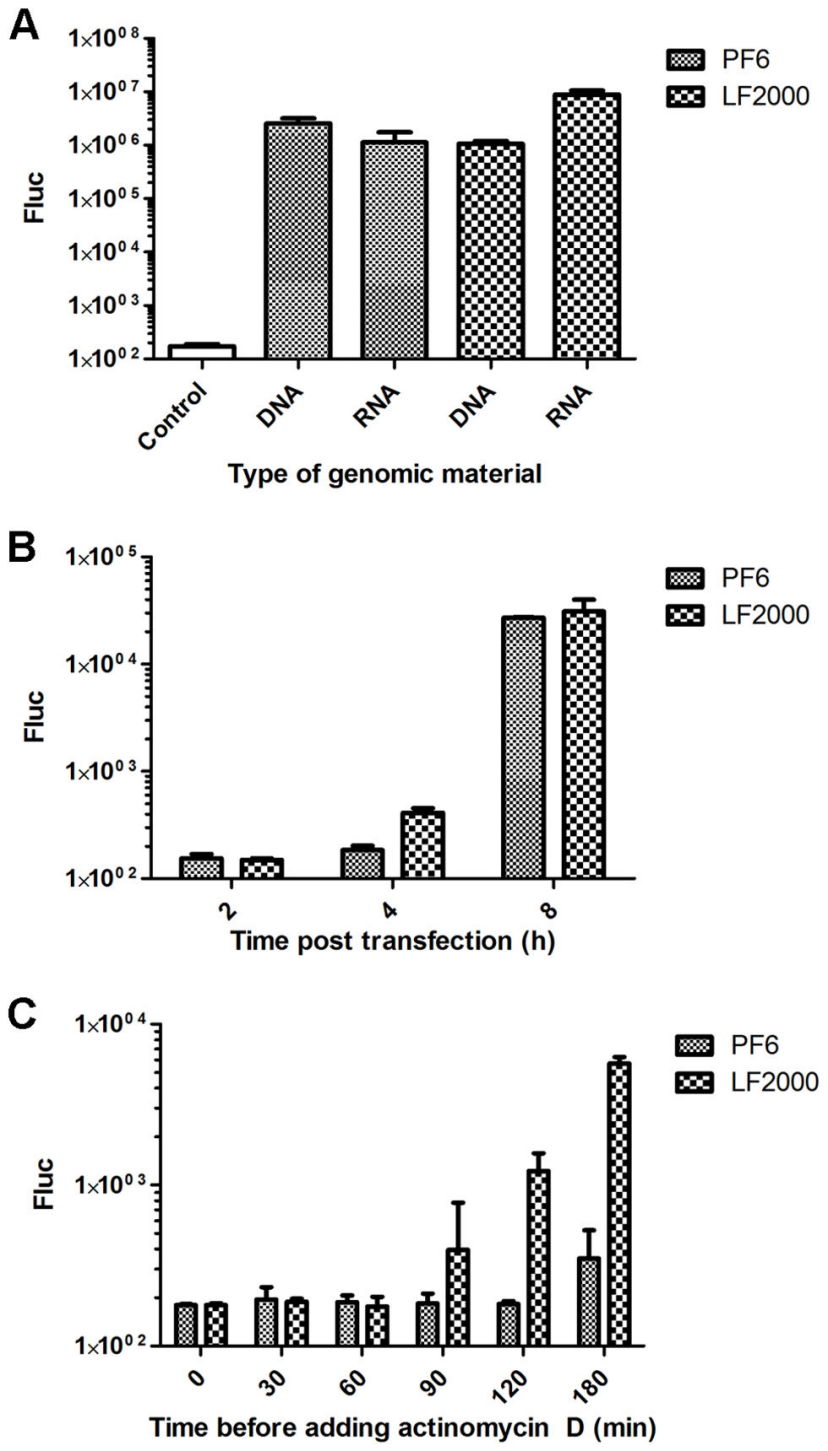
Analysis of Fluc expression in cells transfected with SFV replicon vectors. (A) BHK-21 cells were transfected with 1 µg of pCMV-SFV(Fluc) 1-EGFP DNA or 1 µg of SFV(Fluc) 1-EGFP RNA using the PF6 or LF2000 reagents. Fluc activities were measured at 14 h post transfection. (B) BHK-21 cells were transfected with 1 µg of pCMV-SFV(Fluc) 1-EGFP DNA using the PF6 or LF2000 reagents. Fluc activities were measured at 2 h, 4 h or 8 h post transfection. (C) BHK-21 cells were transfected with 1 µg of pCMV-SFV(Fluc) 1-EGFP DNA using the PF6 or LF2000 reagents. Actinomycin D (final concentration, 20 µg/ml) was added to the transfection medium immediately (“0” time point) or at time points indicated on the horizontal axes. Fluc activities in transfected cell cultures were measured at 8 h post transfection. In all panels, Fluc activities shown on the vertical axes are normalized to the amount of total protein. Experiments were performed in triplicate, and error bars indicate standard deviations.

Transfection with both RNA transcripts of pSFV(Fluc) 4 and pCMV-SFV(Fluc) 4 DNA/RNA layered vector results in the release of recombinant virus capable of spreading in the infected cell culture, making it difficult to assess the number of initially transfected cells. Similarly, as the cells in such infected cultures are at different stages of viral infection, their morphological changes cannot be easily compared. Therefore, to estimate the transfection efficiencies and analyze the morphology of transfected cells, BHK-21 cells were transfected with SFV replicon vectors (RNA transcripts of pSFV(Fluc) 1-EGFP or pCMV-SFV(Fluc) 1-EGFP DNA/RNA layered vector), which do not produce infectious progeny. The cells were fixed or harvested at 14 h post transfection to provide enough time for the replicons to replicate and produce EGFP in amounts sufficient for detection using a fluorescent microscope. When the percentage of EGFP positive cells (Table 1) and Fluc activities measured in transfected cells (Figure 4A) were compared, it was found that PF6 was slightly more effective for the delivery of DNA-based constructs than for the delivery of RNA transcripts. In this experiment, an opposite trend was observed for LF2000 (Figure 4A Table 1). The comparison of the two reagents also revealed that in contrast to the data from the previous experiment, in this experiment PF6 more efficiently delivered DNA to BHK-21 cells than LF2000 (compare Figure 3A and Figure 4A). This discrepancy is not likely due to the different sizes of the pCMV-SFV(Fluc) 4 and pCMV-SFV(Fluc) 1-EGFP vectors (16 kbp *versus* 13 kbp), but rather is most likely due to the different incubation times (8 h *versus* 14 h) used in different experiments. Therefore, it was hypothesized that the DNA delivered to the cell by the PF6 reagent is released more slowly and reaches the nucleus later than that delivered using the LF2000 reagent. Accordingly, the use of the PF6 reagent results in a delay of RNA replication in transfected cells, in turn reducing the luciferase activity produced by the vector at early (Figure 3A) but not late (Figure 4A) times post transfection.

**Table 1:** Comparison of transfection efficiencies achieved using the LF2000 and PF6 reagents.

**Reagent**	**Vector**	**Nucleic acid**	**Percent of EGFP positive cells 14 h post transfection**
PF6	pCMV-SFV(Fluc)1-EGFP	DNA	17%
LF2000	pCMV-SFV(Fluc)1-EGFP	DNA	7%
PF6	SFV(Fluc)1-EGFP	RNA	7%
LF2000	SFV(Fluc)1-EGFP	RNA	15%

Two experiments were performed to verify this hypothesis. First, BHK-21 cells were transfected by pCMV-SFV(Fluc) 1-EGFP using PF6 and LF2000 reagents, and the Fluc activities were measured at 2 h, 4 h and 8 h post transfection. No Fluc expression was detected at 2 h post infection. However, consistent with the proposed hypothesis, at 4 h post transfection the Fluc activity was higher in LF2000 transfected cells, and that difference was lessened at 8 h time point (Figure 4B). Second, we took advantage of the fact that release of self-replicating RNAs from pCMV-SFV(Fluc) 1-EGFP requires transcription by nuclear RNA polymerase II, which is sensitive to actinomycin D. In contrast, the subsequent replication of these RNAs which takes place in cytoplasm is carried out by viral replicase and cannot be suppressed by this inhibitor. Therefore, the beginning of the production of self-replicating viral RNAs can be accurately detected. A corresponding experiment revealed that the addition of actinomycin D at transfection or 30 or 60 min post transfection completely blocks viral RNA production (Figure 4C), indicating that it takes at least 60 min for the transfected plasmid to reach the nucleus and become transcribed. The presence of self-replicating RNAs (judged from Fluc expression) in cells transfected using the LF2000 reagent became evident at 90 min post transfection; in cells transfected using the PF6 reagent, such RNAs were only detected at 180 min post transfection (Figure 4C). These data clearly confirm that DNA delivered to the cell by the PF6 reagent is released more slowly, reaching the nucleus and becoming transcribed approximately 90 min later than DNA delivered using the LF2000 reagent.

### The effects of transfection procedures on the replication of delivered SFV vectors

**Figure 5:**
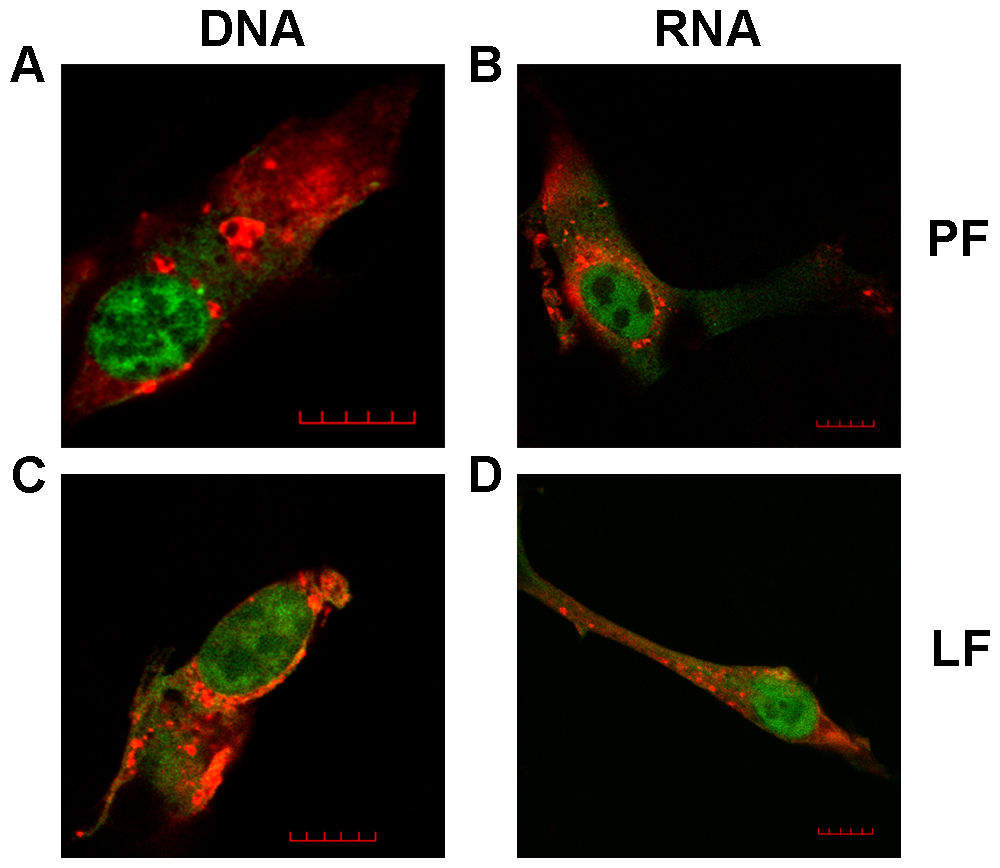
Localization of SFV replication organelles in transfected BHK-21 cells. BHK-21 cells transfected with SFV replicon vectors expressing EGFP and Fluc were fixed at 14 h post transfection. nsP3 of SFV (red on the image) was stained using anti-nsP3 polyclonal antiserum and Alexa 560 conjugated anti-guinea pig antibody; EGFP was detected by its green fluorescence. (A) Cell transfected with pCMV-SFV(Fluc) 1-EGFP DNA using the PF6 reagent. (B) Cell transfected with RNA transcripts from pSFV(Fluc) 1-EGFP using the PF6 reagent. (C) Cell transfected with pCMV-SFV(Fluc) 1-EGFP DNA using the LF2000 reagent. (D) Cell transfected with RNA transcripts from pSFV(Fluc) 1-EGFP using the LF2000 reagent. One characteristic cell is shown in each panel; scale bar represents 10 µm.

To assess the morphology of transfected cells and the presence of virus-specific replication organelles, the cells transfected with RNA transcripts of pSFV(Fluc) 1-EGFP or pCMV-SFV(Fluc) 1-EGFP DNA/RNA layered vectors were analyzed by fluorescence microscopy. Regardless of the construct and transfection reagent used, at 14 h post transfection, EGFP was distributed in both the cytoplasm and the nucleus (Figure 5). Furthermore, immunostaining of the cells for nsP3 of SFV revealed the presence of characteristic cytopathic vacuoles in the cytoplasm, representing the replicase organelles of the SFV [24]. Thus, all analyzed cells had the expected appearance, indicating that neither of the transfection reagents induced aberrations from the normal phenotype. However, note that for BHK-21 cells, 14 h post transfection corresponds to the late stage of SFV infection at which cells are already seriously damaged by the virus. Furthermore, the effects of transfection reagents on replicase organelle formation, which occurs earlier in infection, could not be analyzed using this time point.

### Effects of previous transfection of cells with siRNAs on subsequent SFV infection

Entry and the establishment of replication represent important steps in viral infection. Therefore, it is also important to identify host factors involved in these processes and analyze their effects. The most prominent method for conducting such studies is genome-wide siRNA knockdown based screening, in which cells are transfected with libraries of siRNAs and subsequently infected with viruses. Such studies have indicated large sets of cellular proteins that are presumably involved in the infection of many medically important viruses [25–28]. However, the intrinsic problem for such assays is the surprisingly low reproducibility of the results, with essentially similar assays carried out in different laboratories tending to result in rather different sets of revealed host factors [29]. One possible reason for this phenomenon is that different laboratories use different transfection procedures (and reagents) for siRNA delivery. Unfortunately, several transfection reagents have prominent effects on infection by different viruses (our unpublished observation) on their own and in combination with siRNAs (or other transfected nucleic acids). These non-specific effects can then mask or modify the true effects of knockdown of targeted gene expression on viral infection. Thus, in cases where another molecule (such as siRNA) is introduced into the host cell prior to viral infection, it is critical that the transfection reagent does not affect the ability of the virus to interact with the cells. Therefore, we analyzed whether pre-transfection of the cells by siRNAs targeting the mRNA of EGFP using LF2000 or PF6 reagents influences the ability of SFV to subsequently infect and replicate in such cells.

**Figure 6:**
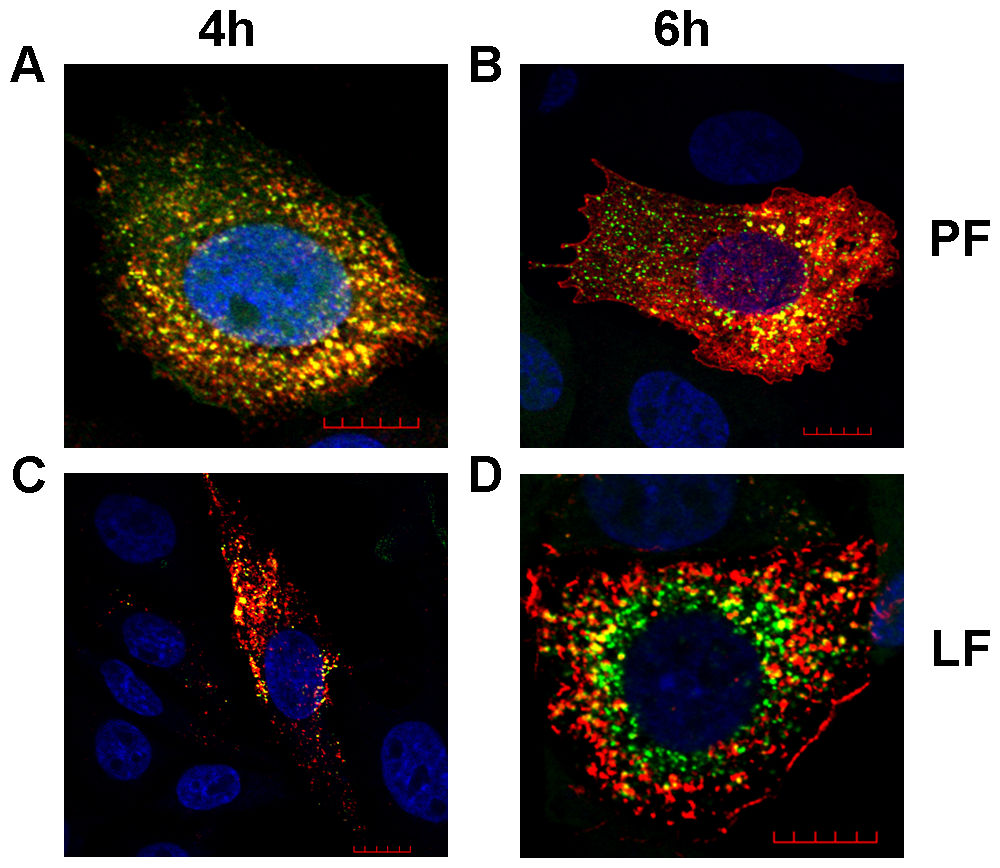
Prior transfection with anti-EGFP siRNAs does not affect SFV infection at 24 h post transfection. CHO-EGFP cells were transfected with siRNA targeting EGFP mRNA using PF6 (A, B) or LF2000 (C, D) reagents. 24 h post transfection, cells were infected with SFV(ZsGreen) 4 at an MOI of 0.1 and fixed at 4 h (A, C) or 6 h (B, D) post infection. The localization of nsP1 (shown in red) of SFV was revealed using rabbit polyclonal antiserum as the primary detection reagent and Alexa 568 conjugated anti-rabbit antibody as the secondary antibody; ZsGreen was detected by its green fluorescence; the co-localization of these signals in virus replication organelles is shown as yellow. Nuclei were counter-stained with DAPI (blue). Images were collected using a LSM 710 confocal microscope (Zeiss); scale bar represents 10 µm. Each panel shows a single optical slice from one characteristic infected cell, and the nuclei of several non-infected cells are also visible.

To study this question, we took advantage of CHO-EGFP cells stably expressing EGFP and pre-transfected them with siRNA: PF6 or siRNA: LF2000 complexes. The CHO-EGFP cells were chosen because such gene knockdown has been previously well characterized [19] and because the effect of siRNA transfection is easy to detect. At 24 h post transfection when the levels of EGFP were already greatly diminished, the transfected cells were infected with SFV(ZsGreen) 4 (Figure 1E). ZsGreen, expressed by SFV(ZsGreen/Xho) 4, is fused to nsP3 and colocalizes with SFV replicase proteins in virus replication organelles, which appear as bright dots in infected cells. In addition, the nsP3-ZsGreen fusion protein also forms different complexes that do not contain other replicase proteins [30]. In contrast, EGFP exhibits diffuse localization. Cells were fixed at 4 h or 6 h post infection and stained with anti-SFV nsP1 antibodies, revealing SFV replicase organelles by the colocalization of ZsGreen and nsP1. In addition, we detected complexes formed by an individual nsP3-ZsGreen molecules and an individual nsP1, which localizes in the plasma membrane [31]. The analysis revealed that at 4 h post infection, the SFV replication organelles appeared to be separated from the plasma membrane and spread across the cell (Figure 6A, 6C); these events follow the established pattern of SFV infection [32]. The overall number and sizes of SFV replication organelles were similar in cells transfected with either reagent (Figure 6). Furthermore, the visual appearance of transfected cells did not differ from that of cells infected without pre-transfection (data not shown). At 6 h post infection, characteristic large vesicles representing SFV replication organelles at this stage of infection were localized in the cytoplasm of infected cells along with other complexes of nsP3-ZsGreen, and nsP1 was abundantly detected at the plasma membrane (Figure 6B, 6D). Again, the observed effects and structures fully correspond to those detected in SFV-infected non-transfected cells [32]. No clear differences were observed between cells transfected using the PF6 or LF2000 reagents. The small differences between images in Figure 6B and Figure 6D were not seen in all collected images (data not shown) and thus most likely represent natural variations between different cells; such differences are commonly observed for low MOI infections (our unpublished observation).

**Figure 7:**
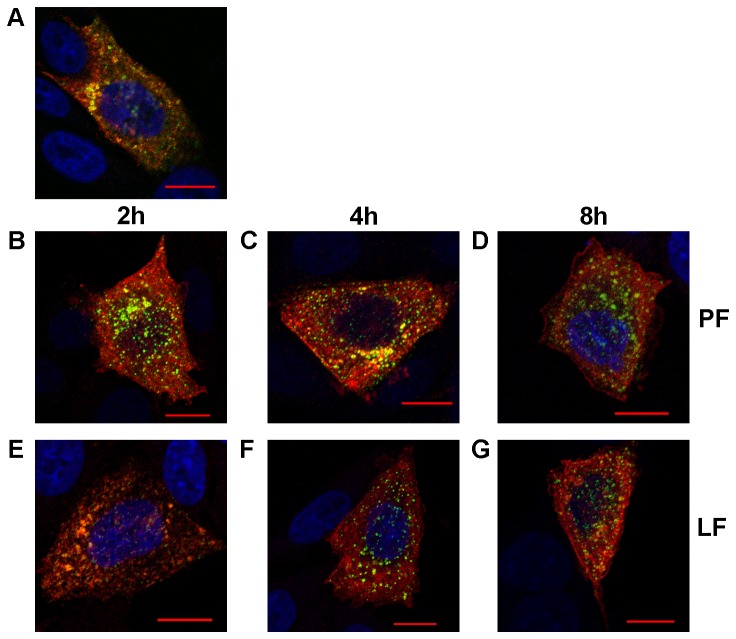
Effects of recent transfection with anti-EGFP siRNA on SFV at 4 h post infection. CHO cells were transfected with siRNA targeting EGFP mRNA using PF6 (B, C, D) or LF2000 (E, F, G) reagents. Transfected cells were infected with SFV(ZsGreen) 4 at an MOI of 0.1 at 2 h (B, E), 4 h (C, F) or 8 h (D, G) post transfection. All cells were fixed at 4 h post infection. The localization of nsP1 (red) of SFV was revealed using rabbit polyclonal antiserum as the primary detection reagent and Alexa 568 conjugated anti-rabbit antibody as the secondary antibody; ZsGreen was detected by its green fluorescence; the co-localization of these signals is shown as yellow. Nuclei were counter-stained with DAPI (blue). Images were collected using a LSM 710 confocal microscope (Zeiss); scale bar represents 10 µm. A single optical slice from a characteristic infected cell is shown in each panel, and the nuclei of several non-infected cells are also visible. Panel A shows a representative cell from the control experiment (no transfection, cell fixed 4 h after infection with SFV(ZsGreen) 4).

**Figure 8:**
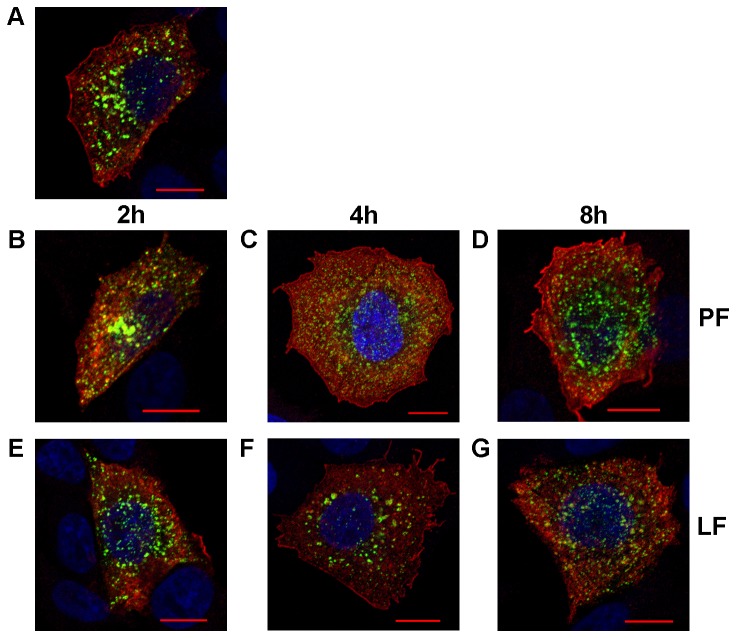
Effects of recent transfection with anti-EGFP siRNA on SFV at 6 h post infection. CHO cells were transfected with siRNA targeting EGFP mRNA using PF6 (B, C, D) or LF2000 (E, F, G) reagents. Transfected cells were infected with SFV(ZsGreen) 4 at an MOI of 0.1 at 2 h (B, E), 4 h (C, F) or 8 h (D, G) post transfection. All cells were fixed at 6 h post infection. The localization of nsP1 (red) of SFV was revealed using rabbit polyclonal antiserum as the primary detection reagent and Alexa 568 conjugated anti-rabbit antibody as the secondary antibody; ZsGreen was detected by its green fluorescence; and the co-localization of these signals is shown as yellow. Nuclei were counter-stained with DAPI (blue). Images were collected using a LSM 710 confocal microscope (Zeiss); scale represents 10 µm. Each panel shows a single optical slice from a characteristic infected cell, and the nuclei of several non-infected cells are also visible. Panel A shows a representative cell from the control experiment (no transfection, cell was fixed at 4 h after infection with SFV(ZsGreen) 4).

Thus, transfection of cells 24 h prior to SFV infection with either LF2000 or PF6 did not result in a consistent effect on the subsequent SFV infection. The experiment was repeated to determine whether this is also the case when the interval between transfection and infection is shorter than 24 h. Normal CHO cells were used to avoid disturbance by the EGFP signal, the quenching of which requires approximately 24 h. In this setup, cells were infected with SFV(ZsGreen) 4 at 2 h, 4 h or 8 h after siRNA transfection and fixed at 4 h (Figure 7) or 6 h (Figure 8) post infection.

The non-transfected control cells fixed at 4 h (Fig. 7A) post infection demonstrated the expected phenotype, with SFV replication organelles that have formed and moved to the cytoplasm of the cell. Cells previously transfected using PF6 showed a similar phenotype as control cells regardless of the time interval between transfection and infection (Fig. 7B, 7C, 7D). In contrast, cells pre-transfected using LF2000 exhibited a notably different phenotype. Furthermore, the observed differences from non-transfected control cells depended on the interval between siRNA transfection and SFV infection. In cells infected at 2 h post transfection, the number of virus replication organelles was clearly reduced (Fig. 7E); this effect decreased when the interval between transfection and infection was increased to 4 h (Fig. 7F) or 8 h (Fig. 7G), but did not completely disappear. Thus, in contrast to the PF6, prior transfection using LF2000 affects the early stages of SFV replication, reducing the efficiency of replication organelle formation and internalization. When pre-transfected cells were analyzed at 6 h post infection (Fig. 8), the cells treated with siRNA: PF6 complexes (Fig. 8B, 8C and 8D) again showed a phenotype that was indistinguishable from that of un-treated control cells (Fig. 8A). At this time point, the phenotypes of cells previously transfected using LF2000 (Fig. 8E, 8F, 8G) were also rather similar to those of the control and PF6 transfected cells. Compared with earlier time point the numbers of virus-induced replicase organelles were increased in these cells (for example, compare Fig. 7E and Fig. 8E). The replicase organelles detected in LF2000 transfected cells at 6 h post infection were somewhat smaller than those in non-transfected control cells (compare Fig. 8A with Fig. 8E, 8F and 8G), but these differences were hardly significant. Taken together, these results suggest that while both the PF6 and LF2000 reagents can be successfully used for pre-transfection of cells prior to infection with alphaviruses, the requirements for the time interval between transfection and infection are different. Use of LF2000 demands a time interval longer than 8 h, and an interval as long as 24 h may be needed. In the case of PF6, even the shortest time interval used in this study (2 h) resulted in no adverse effects on the early stages of SFV infection, indicating that PF6 is a suitable transfection reagent even for applications where transfection must be almost immediately followed by viral infection.

## Discussion

The use of genetically modified viral vectors for basic research and for medical or biotechnological purposes requires an efficient non-viral transfection system to produce a functioning virus from nucleic acid based constructs. In most cases, especially in studies of the inhibitors and host factors of viral infection, the transfection reagent must not affect host cells in a way that alters the course of the viral infection. This study analyzed the ability of the novel peptide-based PF6 reagent to meet these requirements. PF6 has been previously shown to be capable of transporting small nucleic acid molecules such as siRNAs and oligonucleotides into cells [18,19]. PF6 has not been used to transport viral RNA genomes and DNA/RNA layered vector plasmids, which are considerably larger (13-19 kb) and thus more difficult to deliver [33]. Therefore, it was important to determine the experimental conditions for BHK-21 and MEF cells that would allow for successful transfection but would not affect the further replication of the rescued viral genome. The optimal charge ratios of nucleic acid to PF6 reagent for transfecting BHK-21 cells with RNA and DNA were 1:3 and 1:5, respectively, and 1:5 was optimal for both DNA and RNA transfection into MEF cells. Nevertheless, transfection also occurred using other charge ratios (Figure 2B, 2C). Importantly, these non-optimal ratios resulted in efficient transfection, which is a useful property for a transfection reagent, as small variations introduced during the preparation of the transfection mixture will not result in drastic drops in transfection efficiency. In contrast, a large deviation from the optimal ratio resulted in a significant drop in transfection efficiency. For the charge ratios 1:20 and 1:40, this was at least in part due to the toxic effect of high PF6 concentrations on the cell.

Our data revealed that PF6 delivery delayed the rescue of the replication competent RNA genome of a virus or replicon from the DNA/RNA layered vector delivered to the BHK-21 cells. Most likely, this indicates that DNA is released from endosomes and/or transported to the cell nuclei later than the same DNA delivered using LF2000; this time differences was approximately 1.5 h (Figure 4C). In contrast, RNA delivered to the cell cytoplasm is capable of beginning its replication almost immediately after its release from endosomes. This phenomenon explains the observed difference between the measured Fluc activity in cells transfected with DNAs and RNAs of replication-competent vectors (compare Figure 3A and Figure 3B). However, note that in MEF cells transfected by PF6, no effects originating from the delay in DNA release are observed (Figure 3C), indicating differences between cell lines or more likely that the effects are masked by some additional factor(s). In this regard, it should be noted that unlike BHK-21 cells, the primary MEF cells have retained their intact innate immune response. The innate immune response plays a critical role in the detection and limitation of processes associated with alphaviral infection. In the context of this study, the immunocompetent cells can detect the rescue and replication of viral RNA, responding by activating the synthesis and secretion of type-I interferons, which prime uninfected nearby cells by activating numerous cellular antiviral defense mechanisms, making these cells less susceptible or even resistant to subsequent SFVinfection [34,35]. Hence, in MEFs the SFV was most likely capable of replicating in cells where it was rescued but was not able to spread efficiently. Thus, primary MEF cells are very difficult to transfect (our unpublished observation), and it is even more difficult to achieve rescue SFV from RNA or DNA constructs in these cells. The fact that rescue was achieved using both transfection reagents and that PF6 performed as well as if not better than LF2000 in the transfection of DNA vectors (Figure 3C) indicates the potential usability of CPP-based transfection reagents for the delivery of viral nucleic acids under *in vivo* conditions. Interestingly, transfection of *in vitro* transcribed RNAs to MEFs using LF2000 resulted in Fluv expression levels more than an order of magnitude higher than those achieved using the PF6 reagent (Figure 3D). *In vitro* synthesized RNA preparations contain many non-capped RNA transcripts with 5’ tri-phosphate structures representing potent ligands for RIG-I, a cytoplasmic receptor triggering the induction of the innate immune response [36]. The observed high levels of SFV replication may indicate that the LF2000 reagent may deliver its RNA cargo into MEF cells in a way that bypasses some sensors of the innate immune response. If so, this may represent an unwanted effect for the *in vivo* application of transfection reagents.

For a transfection reagent to be successful, it must have other qualities besides high transfection efficiency, such as a simple protocol and a rapid transfection. To this end, we demonstrated that both reagents analyzed here were capable of transfecting the cells even if the transfection mixture was immediately removed from the cells, and that almost maximal efficiency was achieved with a transfection period as short as 20 min (Figure 3). Thus, a very short period of time is sufficient for transfection reagent: nucleic acid complexes to enter (or at least attach themselves to) the cells, ultimately leading to replication of a recombinant viral vector. This property is certainly valuable for the transfection of delicate cells which have strict requirements for growth media and cannot survive (or become damaged) when exposed to transfection media for a long period of time. Importantly, we also demonstrated that considerably longer transfection periods can also be used. While these longer periods did not lead to significant increases in transfection efficiency, they did not produce unwanted side effects or lead to reductions in transfection efficiency (Figure 3).

Taken together, our results confirmed that PF6 is a potent vehicle for the delivery of large DNA or RNA molecules to cultivated cell lines as well as to primary cells. Nevertheless, it must be noted that the head to head comparison of efficiencies achieved using LF2000 and PF6 turned out to be complicated. This complexity was especially evident in the case of the delivery of DNA constructs (compare Figure 3A and Figure 4A), where the obtained results depend on the type of DNA constructs (layered DNA/RNA replication competent vectors or replicon vectors), and more importantly, on the time point (h post transfection) at which the transfection efficiency is analyzed. As noted above, this dependence on time results from the delayed release of DNA from DNA: PF6 complexes. This behavior of PF6 is not unique to the transfection of SFV DNA/RNA layered vectors. A delay in expression was also previously noticed (our unpublished data) even when using PF6 to deliver smaller plasmids encoding for Fluc marker. Thus, it can be hypothesized that long (average plasmid size or longer) DNA constructs require more CPP molecules to bind with them, resulting in a slower dissociation of the peptide-DNA complexes. In the case of viral constructs, this delay results in slower transport of the DNA to the nucleus and ultimately in the delay of replication of the viral genome or replicon RNA. In combination, these effects result in lower levels of marker expression at early times post transfection (Figure 4B, 4C) when the rescue of self-replicating RNAs and the beginning of their replication has not been achieved in all transfected cells. In contrast, at later times (16 h, Figure 4A), the rescue of RNA genomes from DNA and their replication has taken place in all such cells. Accordingly, much higher levels of marker expression are observed, and importantly, the percentage of positively transfected cells is higher for the PF6 reagent than for the LF2000 reagent (Table 1). The situation can be assumed to be the same for DNA/RNA layered replication-competent vectors. However, we were not able to confirm this directly because incubation times longer than 8 h resulted in the release of infectious virions from the cells where the virus rescue occurred more rapidly. Consequently, the infection spread in the cell culture, ultimately leading to infection in every BHK-21 cell and thus masking any differences in transfection efficiencies.

The effects of transfection reagents (and the procedures of their use) on the cells can be monitored by cell viability assays as well as by observing the morphology of the transfected cells [19]. In this study, the transfected cells did not exhibit any noticeable aberrances from the normal morphology (with the exception of cells transfected with mixtures with 1:20 and 1:40 charge ratios). The analysis of cell growth using the xCELLigence System confirmed the absence of general negative effects of PF_6_^−^based transfection on BHK-21 cells. However, transfection reagents may also specifically affect the course of virus infection by altering the intracellular environment. Such changes are especially undesirable when viral vectors are used for studies of viral infection cycles, and they can also lead to false data in genome-wide siRNA screens for host factors involved in viral infection. Therefore, we monitored the formation and phenotype of SFV replicase organelles in cells pre-transfected with siRNAs against EGFP mRNA using the LF2000 or PF6 reagents. This analysis revealed that transfection of eukaryotic cells with siRNAs using the PF6 reagent does not have any detectable effect on the formation of SFV replicase organelles regardless of the time interval between transfection and subsequent infection. This useful property can be explained by the fact that the CPP-based PF6 reagent transports its cargo through the cellular plasma membrane and endosomal membranes without altering their functionality and/or composition. This is especially important in the case of alphavirus infections, where both the plasma membrane and endosomal membranes are crucial for the formation of replicase complexes [31,37]. In contrast, if the cells were infected 2 h or 4 h post transfection with LF2000, the infection efficiency was reduced (data not shown), and importantly, the formation of virus replicase organelles at early stages of infection was clearly suppressed (Figure 7). This effect lessened but did not completely disappear when the interval between transfection and infection was increased to 8 h. The 24 h interval was the only case where previous use of the LF2000 reagent did not result in any detectable effects on the virus replicase organelle formation (Figure 6). In this case, the only difference between the LF2000 and PF6 reagents was a lower EGFP background in PF6 transfected cells (data not shown). This indicated that a higher level of siRNA knockdown was achieved using the PF6 reagent, which is in accordance with results previously obtained by El Andaloussi et al. [19]. The high efficiency of siRNA delivery and the absence of adverse effects on subsequent infection represents an important combination. As all positive strand RNA viruses replicate using cellular membranes, it is possible that this phenomenon is not restricted to alphaviruses. If this is the case, then PF6 and similar transfection reagents allow for advanced siRNA-based screening by recording delicate changes in the phenotypes of infected cells (such as aberrant formation of replicase complexes) in response to the knockdown of host factor expression. Efficient siRNA transfection without affecting virus replication would be especially useful for studies of chronic or persistent infections where cells are infected with virus prior the siRNA transfection. Such approach will facilitate the identification of host factors that are essential at this stage of infection and is useful for the evaluation of the antiviral potency of different siRNA-based inhibitors delivered into chronically infected cells. Taken together, our results indicate that PF6 is an efficient transfection reagent both for the transfection of different cells with viral RNAs and DNA/RNA layered vectors as well as for pre-transfection of cells that are subsequently infected by viruses with siRNAs or potentially other nucleic acids.

## Conclusions

Various methods have been developed to transport nucleic acids into eukaryotic cells. These methods can generally be divided into two distinct categories – viral and non-viral methods. Viral methods rely on delivery pathways that are specific for each virus. It is generally assumed that viruses attach themselves to cellular receptors, resulting in cell entry, although in the case of some well-studied viruses these processes show both redundancy and complexity. Some non-viral reagents such as cationic lipids utilize similar mechanisms of internalization, while some methods affect the cell physically (electroporation, microinjection). However, none of these methods are perfect for use in *in vitro* cell culture and even less so in significantly more complex *in vivo* systems. Here, we studied the possibility of combining novel transfection reagent PF6, which has been previously shown to transfect both smaller and larger nucleic acid molecules, with SFV replication-competent and replicon vector technology. We first demonstrated the usability of PF6 for transfection of cells with such vectors. The transfection was rapid, simple and did not damage the host cells on its own. All cell cultures transfected with the replication competent vector become infected, indicating the release of the infectious virus. Second, we documented that while PF6 achieves highly efficient delivery of viral DNA constructs, the release of self-replicating RNA from these DNAs took longer than for the LF2000 reagent. Finally, we demonstrated that pre-transfection of the cells with siRNAs using PF6 reagent has no adverse effects on the subsequent SFV infection, which was not the case for LF2000. These results show that these two agent are essentially equipotent for the rescue of the RNA genome of the virus from nucleic acids under *in vitro* conditions. It remains unknown how this could be translated to an *in vivo* situation, as LF2000 is not designed for such a purpose and an examination of this question was beyond the scope of the current study. Importantly, for applications where cells must be pre-transfected with siRNAs and/or other nucleic acids prior to the infection with alphavirus (and possibly other positive strand RNA viruses), the PF6 is the reagent of choice due to its high efficiency of siRNA transfection and to the absence of adverse effect on cells and on subsequent viral replication.
